# Gut microbiota and inflammation patterns for specialized athletes: a multi-cohort study across different types of sports

**DOI:** 10.1128/msystems.00259-23

**Published:** 2023-07-27

**Authors:** Yuxue Li, Mingyue Cheng, Yuguo Zha, Kun Yang, Yigang Tong, Song Wang, Qunwei Lu, Kang Ning

**Affiliations:** 1 Key Laboratory of Molecular Biophysics of the Ministry of Education, Hubei Key Laboratory of Bioinformatics and Molecular Imaging, Center of Artificial Intelligence Biology, College of Life Science and Technology, Huazhong University of Science and Technology, Wuhan, China; 2 Department of Bioinformatics and Systems Biology, College of Life Science and Technology, Huazhong University of Science and Technology, Wuhan, China; 3 Exercise Immunology Center, Wuhan Sports University, Wuhan, China; 4 College of Life Science and Technology, Beijing University of Chemical Technology, Beijing, China; Pontificia Universidad Catolica de Chile, Santiago, Chile

**Keywords:** multi-sport, gut microbiota, inflammation, latent Dirichlet allocation, aerobics, wrestling, rowing

## Abstract

Regular high-intensity exercise can cause changes in athletes’ gut microbiota, and the extent and nature of these changes may be affected by the athletes’ exercise patterns. However, it is still unclear to what extent different types of athletes have distinct gut microbiome profiles and whether we can effectively monitor an athlete’s inflammatory risk based on their microbiota. To address these questions, we conducted a multi-cohort study of 543 fecal samples from athletes in three different sports: aerobics (*n* = 316), wrestling (*n* = 53), and rowing (*n* = 174). We sought to investigate how athletes’ gut microbiota was specialized for different types of sports, and its associations with inflammation, diet, anthropometrics, and anaerobic measurements. We established a microbiota catalog of multi-cohort athletes and found that athletes have specialized gut microbiota specific to the type of sport they engaged in. Using latent Dirichlet allocation, we identified 10 microbial subgroups of athletes’ gut microbiota, each of which had specific correlations with inflammation, diet, and anaerobic performance in different types of athletes. Notably, most inflammation indicators were associated with *Prevotella*-driven subgroup 7. Finally, we found that the effects of sport types and exercise intensity on the gut microbiota were sex-dependent. These findings shed light on the complex associations between physical factors, gut microbiota, and inflammation in athletes of different sports types and could have significant implications for monitoring potential inflammation risk and developing personalized exercise programs.

**IMPORTANCE:**

This study is the first multi-cohort investigation of athletes across a range of sports, including aerobics, wrestling, and rowing, with the goal of establishing a multi-sport microbiota catalog. Our findings highlight that athletes’ gut microbiota is sport-specific, indicating that exercise patterns may play a significant role in shaping the microbiome. Additionally, we observed distinct associations between gut microbiota and markers of inflammation, diet, and anaerobic performance in athletes of different sports. Moreover, we expanded our analysis to include a non-athlete cohort and found that exercise intensity had varying effects on the gut microbiota of participants, depending on sex.

## INTRODUCTION

Regular physical exercise is beneficial for human health, such as optimizing cardiorespiratory fitness, immunity, insulin sensitivity, and body composition (1, 2). This is especially true for athletes, who focus on fitness-enhancing training to improve their athletic performance. Previous studies have found significant changes in the composition and function of the gut microbiome in athletes under the influence of prolonged high-intensity physical exercise (2 - 4). For instance, compared to normal people, rugby players had a higher abundance of Firmicutes and lower levels of Bacteroidetes (5). Marathon runners had an increased presence of *Veillonella* (6). Professional cyclists had an increased abundance of *Methanobrevibacter smithii* and *Prevotella* (7). These suggested that different types of athletes with specialized training might have different gut microbial variations compared to healthy individuals. Specialized gut microbial changes can affect athletic performance through multiple avenues, such as regulating excitatory and inhibitory neurotransmitters to alleviate psychological stress (8, 9), and converting exercised-induced lactate into propionate to improve endurance (6). However, it is unclear how the gut microbiota profiles of athletes differ across different types of sports and whether specialized microbial patterns are associated with specific training models. A better understanding of these links could deepen our understanding of the specialized microbial profiles under specific training patterns, thereby facilitating the development of personalized exercise modulation for individuals.

However, when physical activity is too intense, athletes may experience negative effects of gut microbiota changes during exercise (10), such as gut inflammation. Gut inflammation was correlated with an increased risk of viral or bacterial infection, partially caused by excessive physical activity (11). Previous studies have found that physical activity resulted in profound differences in inflammatory and metabolic markers between professional athletes and controls (12). For example, rugby players exhibit lower levels of proinflammatory cytokines (12), and endurance athletes show increased production of butyrate (a modulator of proper immune function) (13). Changes in inflammation-related factors in athletes might imply different degrees of potential gut inflammation risk. However, few studies have focused on microbes’ correlations with the gut inflammation that athletes may experience during exercise. Exploring the potential of gut microbiota in adjusting the inflammation of athletes might help them to alleviate the effects of sports injuries. Therefore, we constructed a comprehensive multi-cohort study including information about physical fitness, diet, blood measurements, and gut microbial profiles for different types of athletes. We aimed to grasp the specialization of gut microbiota across multiple sports, as well as their associations with inflammation. This information could prove highly significant for monitoring the potential inflammation risk and further achieving personalized exercise modulation.

In this study, we built a microbiome catalog for athletes across different types of sports and answer key questions including how gut microbiota differed in athletes from different types of sports; which microbes were associated with and their potential effects on inflammation, diet, and anaerobic performance; and what were the differences in gut microbiota among participants with different exercise intensities.

## RESULTS

### Overview of the multi-cohort study across different types of sports

**Fig 1:**
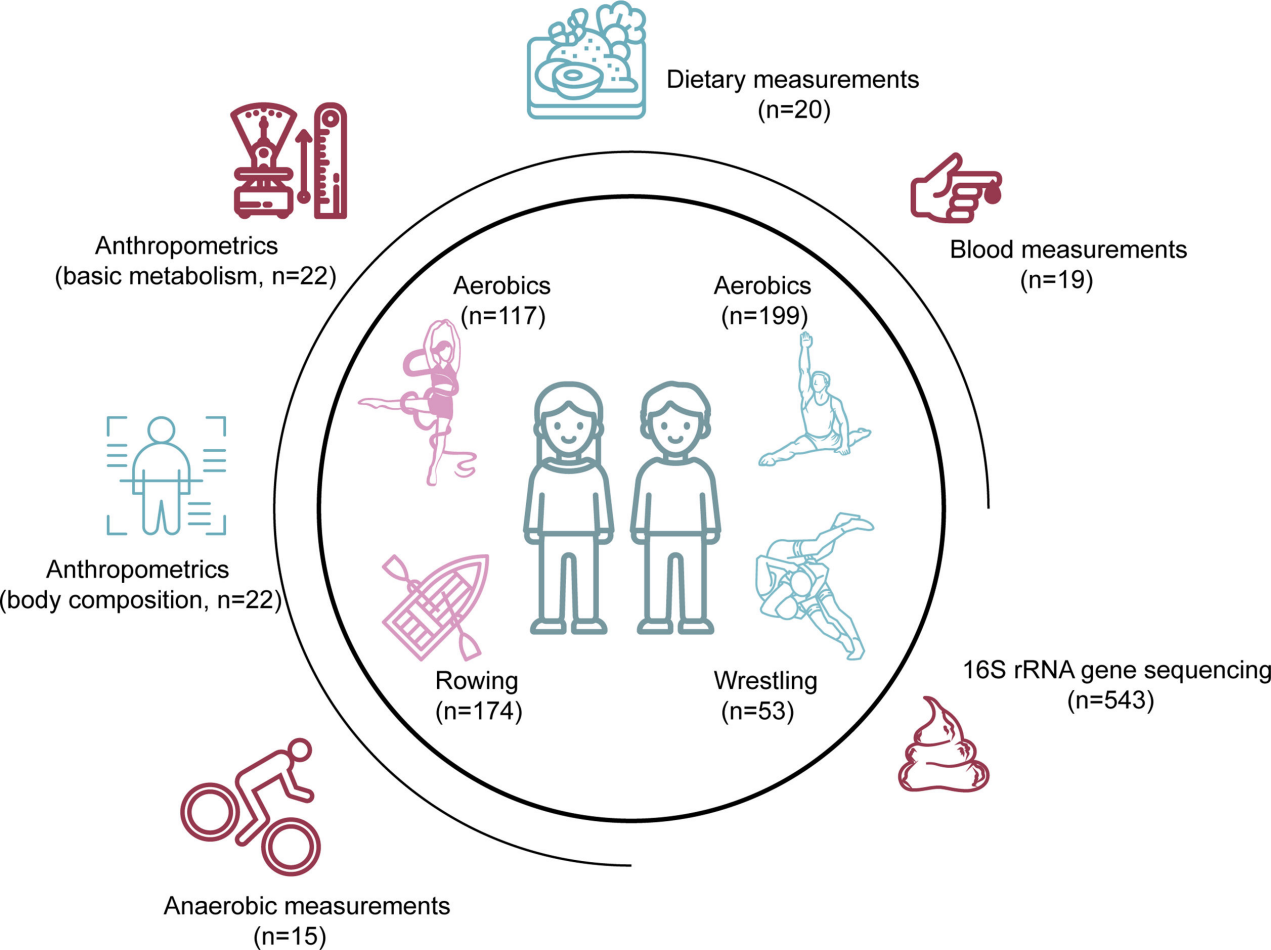
Overview of the multi-sport cohort. Diagram summarizes data cohort and available metadata (*n* = number of variables collected).

We collected a total of 543 fecal samples from athletes in three types of sports, including aerobics (AER), wrestling (WRE), and rowing (ROW), and designated these samples as the multi-sport meta-cohort (MS cohort, Fig. 1). These various sports have specialized training patterns with AER focusing on flexibility, ROW on endurance, and WRE on both endurance and explosive power. Owing to the genetic, physical, and environmental differences between females and males, we divided the MS cohort into two sub-cohorts for the following analysis, comprising the MS-female and MS-male cohorts. The MS-female cohort was composed of 117 AER female athletes and 174 ROW female athletes, while the MS-male cohort was composed of 199 AER male athletes and 53 WRE male athletes. In addition, we collected 19 blood measurements, 20 dietary measurements, 22 anthropometrics (basic metabolism and body composition), and 15 anaerobic measurements to be correlated with athletes' gut microbial profiles (Table S1). Furthermore, to investigate the impact of exercise intensities on gut microbiota, we additionally recruited and examined 178 fecal samples from non-athletes.

### Establishment and comparison of multi-sport gut microbial profiles

**Fig 2:**
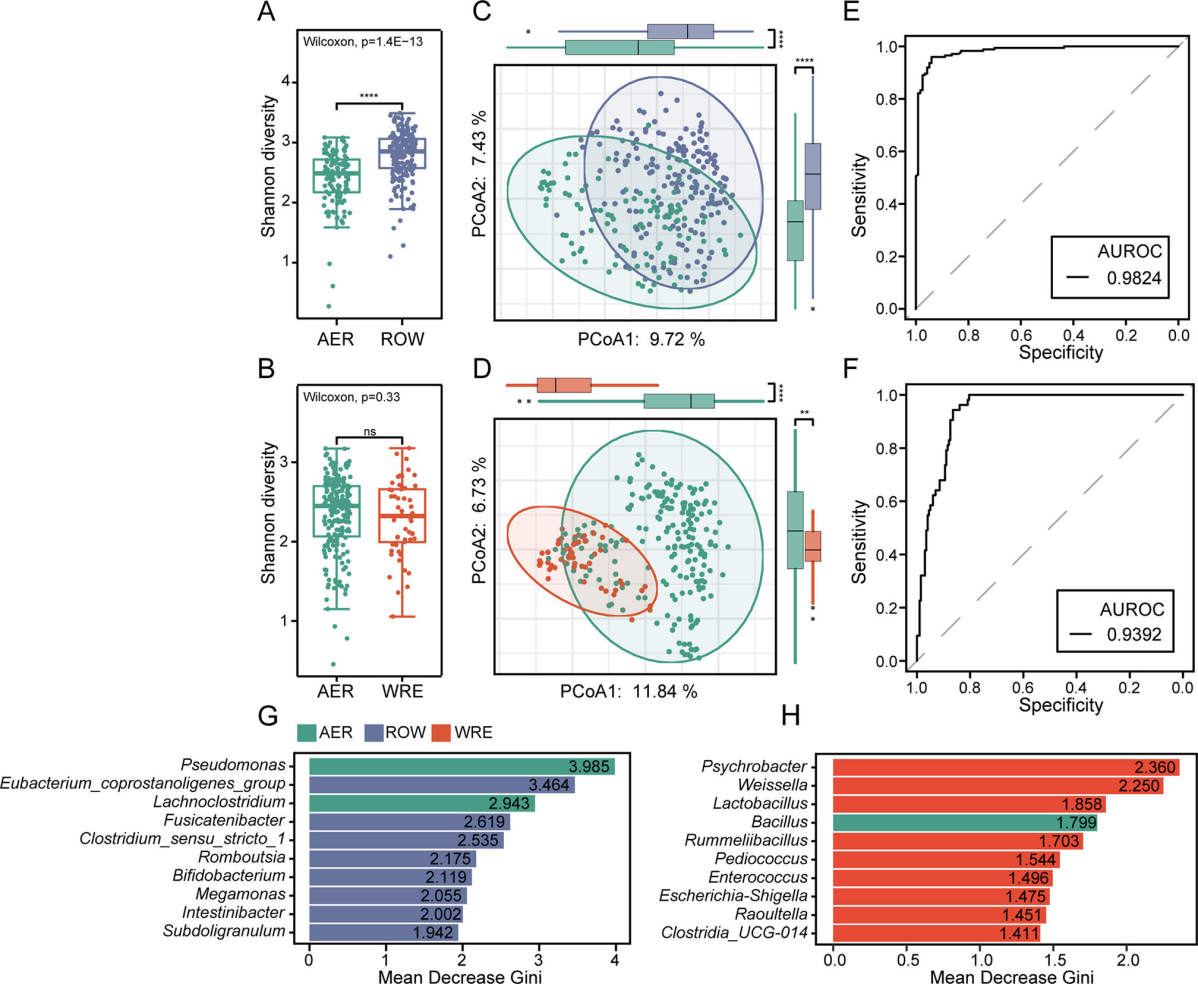
Establishment and comparisons of multi-sport gut microbiota profiles. (A–D) Comparison of the microbial Shannon diversity and microbial overall profiles between different types of sports in the MS-female cohort (A and C), as well as in the MS-male cohort (B and D). Microbial overall profiles were determined by the PCoA using Bray-Curtis differences at genus level (E–F). The ROC curves of the random forest classification in types of sports using microbial genera as features, with AUROC displayed in the MS-female cohort (E) and the MS-male cohort (F). The top 10 genus contributed to the random forest classification in the MS-female cohort (G) and the MS-male cohort (H). **P < 0.05; ****P < 0.001; and ns, not significant. ROC, receiver operating characteristic.

We first established a multi-sport gut microbial profile and found that aerobics, wrestling, and rowing athletes have their specific gut microbial profiles. In the MS cohort, we identified gut microbes at phylum (*n* = 9), class (*n* = 13), order (*n* = 27), family (*n* = 52), and genus (*n* = 111) levels that were presented in more than 10% of all the 543 fecal samples, as the MS microbial catalog. We then compared these gut microbes at each taxonomic level between different types of sports in the MS-female and MS-male cohorts, respectively (Table S2). Phyla Actinobacteriota (*P* = 2.09E−13) and Proteobacteria (*P* = 1.74E−14) were most differently distributed in the MS-female and MS-male cohorts, respectively. We found that the differentiation in gut microbes was notable, with 77% of genera differently distributed between AER and ROW in the MS-female cohort and 64% of genera between AER and WRE in the MS-male cohort (*q* < 0.05). We then investigated the differentiation in microbial diversity. In the MS-female cohort, ROW athletes’ gut microbiota had higher Shannon diversity than AER athletes’ gut microbiota (*P* = 1.4E−13, Fig. 2A), and their overall profiles were obviously separated against the PCoA1 axis (*P* = 1.09E−12) and PCoA2 axis (*P* = 1.42E−14) of principal coordinates analysis (PCoA) based on Bray-Curtis dissimilarities at the genus level (Fig. 2C). The random forest algorithm was applied to these samples and arrived at a receiver operating characteristic (AUROC) of 0.9824 (Fig. 2E), further validating the differentiation of their gut microbial profiles. In the MS-male cohort, the Shannon diversity showed no significant difference between WRE athletes’ gut microbiota and AER athletes’ gut microbiota (Fig. 2B). However, the differentiation in their overall profiles was notable, as demonstrated by their obvious separation in PCoA and a high AUROC of 0.9392 in random forest algorithm (Fig. 2D and F). In addition, we introduced sedentary (SD) populations to the MS cohort as a control to construct a Random Forest model (Fig. S1A through D). Compared with the MS cohort, the area under curve receiver operating characteristic (AUROC) values were reduced in both male and female groups (0.9464 and 0.6846, respectively).

Moreover, Fig. 2G and H showed the top 10 genera with the largest contribution to random forest classification in the MS-female and MS-male cohorts, respectively. In the MS-female cohort, *Pseudomonas* and *Eubacterium_coprostanoligenes*_*group* were the most discriminant features for female AER and ROW samples. In the MS-male cohort, *Psychrobacter* and *Bacillus* were the most discriminant features for male WRE and AER samples. These results suggested that both female and male athletes had specialized gut microbiota for their respective sports.

### Co-occurrence networks for multi-sport gut microbiota

**Fig 3:**
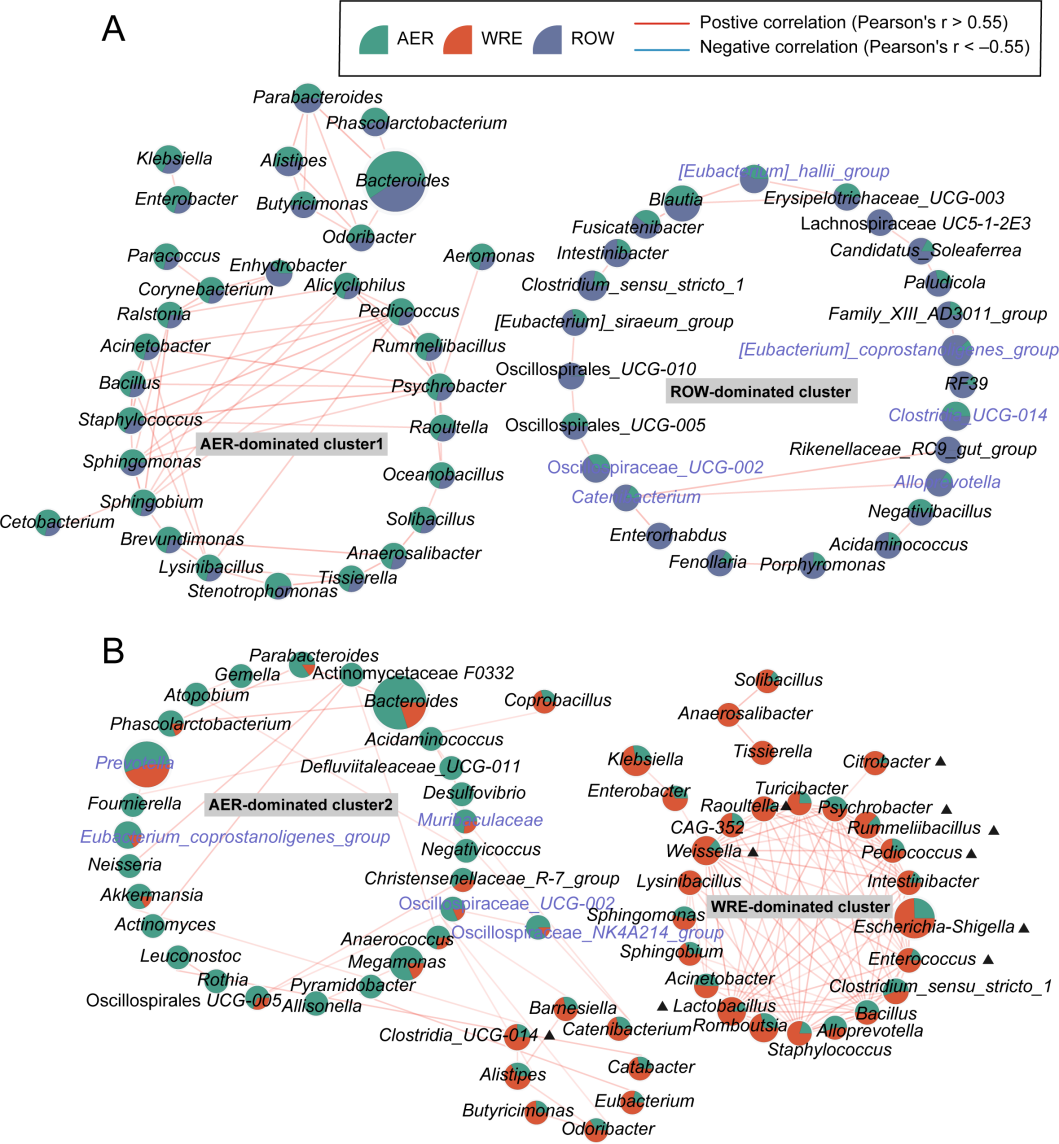
Co-occurrence networks of multi-sport gut microbiota. (A and B) The networks of genera were constructed in MS-female (A) and MS-male cohorts (B), respectively. Each circle represents a genus with the size representing relative abundance and the color representing its enrichment in the group. The edges indicate significant Spearman correlations between genus (*P* < 0.05 and absolute value of SCC > 0.55). Red edges indicate positive correlations, and blue edges indicate negative correlations. SCC, Spearman correlation coefficient.

We next investigated the interaction patterns in the gut microbiota of different types of athletes. In the MS-female cohort (Fig. 3A), we identified two network clusters based on Spearman correlations at the genus level, one of which contained bacteria enriched in the AER group (AER-dominated cluster 1), while the other contained bacteria enriched in the ROW group (ROW-dominated cluster), such as *Eubacterium*_*hallii*_*group* and *Clostridia*_*UCG*-*014*. In the MS-male cohort, we also identified two network clusters including AER-dominated cluster 2 and WRE-dominated cluster (Fig. 3B). Moreover, when SD populations were recruited in the analysis, we identified an SD-dominated cluster, differentiated from those MS clusters (Fig. S1E and F). These findings suggested that different types of populations shared different interaction patterns of the gut microbiota.

### Gut microbial composition patterns resolved by latent Dirichlet allocation

**Fig 4:**
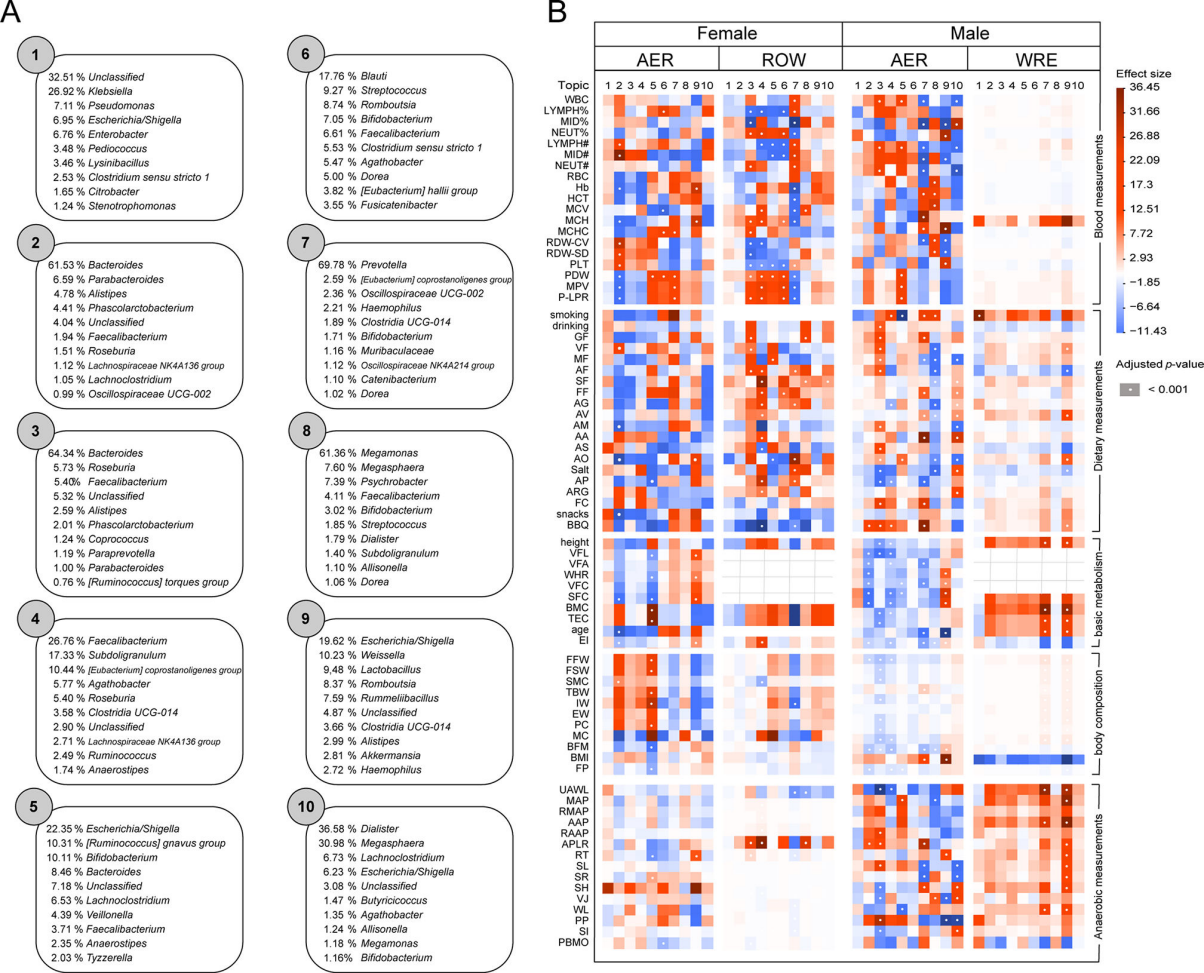
Associations of athletes gut microbial subgroups with blood measurements, dietary measurements, anthropometrics, and anaerobic measurements. (A) Gut microbial subgroups (topics) were identified by LDA. The top 10 genera with the highest probability per topic were displayed. NA represents operational taxonomic units (OTUs) not classified to a genus. The topic number is random and serves only as an identifier. (B) The heatmap shows the associations of athletes gut microbial subgroups with blood measurements, dietary measurements, anthropometrics, and anaerobic measurements. The associations were calculated by Dirichlet regression models. Each association between a topic and measurement was represented by a square, where the intensity of the color indicates the effect size. Red indicates the positive association and blue indicates the negative association. A white dot in the center of a circle indicates that the association remained significant after FDR correction (*q* < 0.001). Gray grid means no data. WBC, white blood cell count; LYMPH%, lymphocyte ratio; MID%, intermediate cell ratio; NEUT%, neutrophil ratio; LYMPH#, lymphocyte count; MID#, intermediate cell count; NEUT#, neutrophil count; RBC, red blood cells; Hb, hemoglobin; HCT, hematocrit; MCV, mean red blood cell volume; MCH, mean erythrocyte hemoglobin; MCHC, mean hemoglobin concentration; RDW-CV, red blood cell distribution concentration; RDW-SD, red blood cell distribution width; PLT, platelet; PDW, platelet distribution; MPV, mean platelet volume; P-LPR, large platelet ratio; GF, grain frequency; VF, vegetables frequency; MF, meat frequency; AF, aquatic frequency; SF, soy frequency; FF, fruit frequency; AG, amount of grain; AV, amount of vegetables; AM, amount of meat; AA, amount of aquatic; AS, amount of soy; AO, amount of oil; AP, amount of pepper; ARG, amount of raw garlic; FC, fruit count; VFL, visceral fat levels; VFA, visceral fat area; WHR, waist to hip ratio; VFC, visceral fat content; SFC, subcutaneous fat content; BMC, basal metabolic capacity; TEC, total energy consumption; EI, electrical impedance; FFW, fat free weight; FSW, fat free soft weight; SMC, skeletal muscle content; TBW, total body water; IW, intracellular water; EW, extracellular water; PC, protein capacity; MC, minerals capacity; BFM, body fat mass; BMI, body mass index; FP, fat percentage; UAWL, upper extremity anaerobic work load; MAP, maximum anaerobic power; RMAP, relative maximum anaerobic power; AAP, average anaerobic power; RAAP, relative average anaerobic power; APLR, anaerobic power lapse rate; RT, reaction time; SL, standing on left leg with eyes closed; SR, standing on right leg with eyes closed; SH, sitting height; VJ, vertical jump; WL, wing length; PP, push-ups 1 min; SI, step index; PBMO, power bike max oxygen uptake; FDR, false discovery rate.

Further, we introduced latent Dirichlet allocation (LDA) to resolve the latent structure of athletes' gut microbiota. LDA, a Bayesian probabilistic generative model (14 - 16), could reduce the dimensionality of the microbial data into subgroups (topics), where microbes within the same topic were modulated by shared latent factors. We identified a total of 10 microbial topics at the genus level to describe each participant’s gut microbiota composition (Table S3). Each topic represented a specific probability distribution of certain genera (Fig. 4A). We found that the topic proportions were different across multi-sport gut microbiota (Fig. S2A and B). Hierarchical clustering of the 10 topics (Fig. S2C) revealed that topics containing the same dominant bacteria (e.g., topics 2 and 3) or containing certain overlapped genera (e.g., topics 8 and 10) were clustered into the same group.

### Associations between gut microbial subgroups and inflammation related to intensive exercise

Next, we investigated the gut microbial subgroups (topics) associated with inflammation. The Dirichlet regression model was performed to test the associations between gut microbial subgroups (topics) and blood measurements (Fig. 4B), and these phenotypic factors had a highly explained variance for microbial diversity and function (Fig. S3). In the ROW female group, topics 3, 4, 5, 6, 7, and 8 were associated with one or more blood measurement factors (*q* < 0.001), and topic 7 exhibited the strongest positive associations. As a result, ROW female athletes with a high probability of topic 7 tended to have higher leukocyte (*P* = 1.99E−13), lymphocyte count (*P* = 9.81E−12), intermediate cell count (*P* = 2.42E−13), and neutrophil count (*P* = 2.76E−08). These factors were common indicators of inflammation in the blood test and varied with different types of sports (Fig. S4A and B). Moreover, we noticed that most genera of topic 7 were reported to be associated with sports inflammation, including *Prevotella*, *Oscillospira*, *Coprococcus*, and *Haemophilus* (Table S4). *Prevotella* (69.78% of topic 7) colonization in the gut resulted in metabolic changes in the microbiota, reduced interleukin 18 (IL-18) production that exacerbated intestinal inflammation, and might lead to systemic autoimmunity. Additionally, *Prevotella* contained enzymes that played an important role in mucus degradation, possibly leading to increased intestinal permeability (17). Previous research has suggested that increased intestinal permeability might favor colonic bacterial translocation, with a consequent risk of gastrointestinal problems (18, 19). In mouse models, exhaustive exercise promoted intestinal inflammation and increased the growth of *Oscillospira*, and *Coprococcus* (20), which also had a high percentage in topic 7. In addition, when evaluated in a post-exercise phase, exhaustive exercise can cause immune function depression, thereby increasing the risk of viral and bacterial infections (11), with *Haemophilus* (2.21% of topic 7) hosting various pathogenic species (21). In general, these genera might play a crucial role in enhancing gut permeability and the risk of infection, promoting inflammatory responses in athletes.

Interestingly, the significant associations between topic 7 and those inflammation factors were also observed in the AER male group but they were negative (Fig. 4B). The AER male group with a high probability for topic 7 was characterized by a low leukocyte (*P* = 1.06E−09), lymphocyte count (*P* = 1.74E−05), intermediate cell count (*P* = 7.33E−37), and neutrophil count (*P* = 5.18E−12). The correlation between topic 7 and inflammation may reflect the collective response of the microbes it encompasses, and we found that *Eubacterium*_*hallii*_*group* (*P* = 4.1E−07), *Clostridia_UCG*-*014* (*P* = 0.015), *Alloprevotella* (*P* = 1.6E−05), and Oscillospiraceae_*UCG*-*002* (*P* = 7.0E−08) were more enriched in the ROW female group, while *Prevotella* (*P* = 0.006) was more enriched in the AER male group (Fig. 3 and Fig. S4C). Therefore, the difference in correlation orientation might result from the different enriched genera of topic 7 between the female ROW and male AER groups. We further performed a regression analysis of these bacteria and inflammatory indicators (Fig. S4D and E). For the ROW female group, *Clostridia*_*UCG*-*014* and *Eubacterium*_*coprostanoligenes*_*group* were consistently negatively correlated with leukocyte (*P* = 0.00023, *P* = 9.6E−07) and neutrophil count (*P* = 3E−05, *P* = 5.1E−07). In AER male group, it can be observed that *Alloprevotella* and *Prevotella* were consistently negatively correlated with leukocyte (*P* = 0.012, *P* = 0.0052) and lymphocyte count (*P* = 0.0012, *P* = 0.047), while AER female did not observe a significant correlation with inflammation. Overall, these results revealed that certain microbes enriched in topic 7 were associated with inflammation, and this association was influenced by sports’ types. Differences in the abundance of microbes in hosts with different sport types or sex may lead to specialized inflammation patterns, such as *Eubacterium*_*coprostanoligenes*_*group, Clostridia*_*UCG*-*014* were correlated with the inflammation of female rowing athletes.

### Associations of gut microbial subgroups with dietary information and anaerobic performance

Moreover, the Dirichlet regression model showed that most associations of gut microbial subgroups (topics) with dietary measurements and anthropometrics measurements were observed in the AER male group (Fig. 4B). Topic 3 was positively associated with food intake including drinking, vegetable frequency, meat frequency, aquatic frequency, and barbecue (BBQ; *q* < 0.0005). Meanwhile, topic 3 was negatively correlated with fat free weight, skeletal muscle content, and body fat mass (*q* < 0.0001). The results suggested that topic 3 might be the key microbial subgroup in inhibiting obesity under high caloric intake. Topic 2 was found to be strongly negatively correlated with anthropometrics (basic metabolism) factors such as visceral fat levels, waist to hip ratio, and subcutaneous fat content (*q* < 0.0001), indicating a potential role in weight control. Notably, topics 2 and 3 belonged to the same topic cluster and shared similar genera probability distributions (Fig. S2D and E). *Phascolarctobacterium* has a high proportion in both topics, and numerous studies reported that it was inversely associated with obesity (22). In general, both topics 2 and 3 might play a crucial role in controlling the weight of athletes.

We then explored the associations between gut microbial subgroups and anaerobic measurements. Topic 9 had the strongest positive association with anaerobic measurements such as upper extremity anaerobic work load, maximum anaerobic power, and average anaerobic power (*q* < 2.48E−5) in the WRE male group. This indicated that topic 9 might have a potential role in enhancing anaerobic performance, which warranted further investigations.

### Sex-dependent different intensities of exercise affect the human gut microbiota

**Fig 5:**
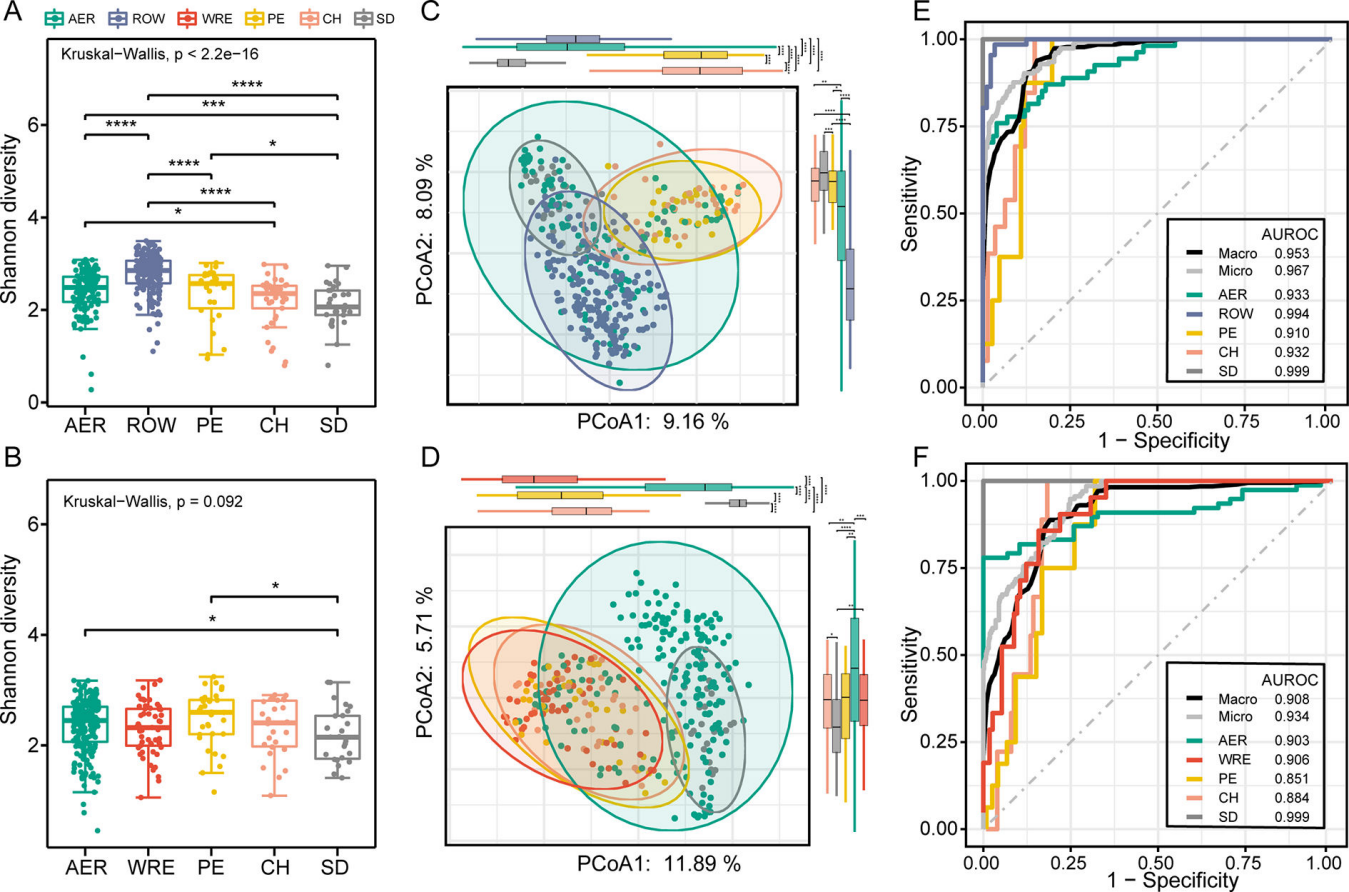
Effects of different degrees of exercise on human gut microbiota. (A) The boxplots show the microbial Shannon diversity among five female groups including AER, ROW, PE, CH, and SD. (B) The boxplots show the microbial Shannon diversity among five male groups including AER, WRE, PE, CH, and SD. (C and D) PCoA of female participants (C) and male participants (D) based on the Bray-Curtis differences. (E and F) The ROC curves of the random forest classification in five groups using microbial genera as features, with AUROC displayed in the female groups (E) and the male groups (F). *P < 0.1; **P < 0.05; ***P < 0.01; ****P < 0.001; ROC, receiver operating characteristic.

Finally, we sought to investigate the effects of different exercise intensities on gut microbiota. To this end, we introduced three additional non-athlete cohorts, including physical major students (PE), children (CH), and sedentary people (SD). We found Shannon diversity was also affected by exercise intensity. For females, we found that athletes had higher microbial Shannon diversity than that non-athletes (*P* < 0.05). Especially, the ROW group had the highest Shannon diversity of all the other four groups (*P* = 2.86E−22, Fig. 5A). However, for males, the differences in Shannon diversity were not significant between athletes and non-athletes groups (*P* = 0.092, Fig. 5B). And the gut microbial compositions could be differentiated by exercise intensity for both males and females (*P* < 0.05, Fig. 5C and D). Moreover, the Random Forest model showed that female groups had higher classification accuracy when discriminating one group out of the five groups (AUROC_AER_ = 0.933, AUROC_ROW_ = 0.994, AUROC_PE_ = 0.910, AUROC_CH_ = 0.932, AUROC_SD_ = 0.999, Fig. 5E), as compared with that of the male groups (AUROC_AER_ = 0.903, AUROC_WRE_ = 0.906, AUROC_PE_ = 0.851, AUROC_CH_ = 0.884, AUROC_SD_ = 0.999, Fig. 5F). These results suggested that exercise intensity had effects on the gut microbiota, and these effects were sex-dependent.

To further assess the effect of sex-dependent exercise intensity on gut microbiota, we then identified the top 5 genera with the highest importance in the Random Forest model (quantified by the mean decrease accuracy) for each group (Fig. S5). We found that the abundance of *Clostridium*_*sensu_stricto*_*1* decreased as the female participants’ exercise intensity decreased, especially in athletes compared to SD, while a similar trend was observed for *Intestinibacter* in male participants (Fig. S5). In addition, we investigated the distribution of inflammation-associated microbiota (the microbiota in topic 7) in each cohort. *Prevotella* as the dominant microbe in topic 7 (69.78% of topic 7) was found to decrease as the intensity of exercise decreased, especially in male participants (Fig. S6). Taken together, certain gut microbial alterations could respond to the different intensities of exercise, and sex was a non-negligible factor in both microbial changes and the potential inflammation risk.

## DISCUSSION

Athletes’ gut microbial profiles were influenced by the types of sports they participated in. Previous studies have demonstrated that exercise could increase microbial diversity in the gut microbiome (12, 23). In the present study, the microbial diversity of the ROW female group was profoundly higher than AER female group, suggesting that different types of exercise have different effects on microbial diversity in female athletes, but no significant differences were observed between male athletes. The analysis of microbial composition and diversity of different types of athletes revealed that the gut microbial community of different types of athletes differs profoundly. These results confirmed that the pattern, intensity, and frequency of physical exercise cannot be ignored when studying the effects of physical activity on the microbial community. In other words, to monitor the potential risk of inflammation and further realize individualized exercise modulation, it is necessary to consider different types of athletes when studying the association between microbiota and inflammatory factors.

The associations between gut microbiota and blood measurements confirmed that inflammatory factors were affected by the types of sports. Blood measurements showed the strongest and most consistent associations with topic 7 in ROW female and AER male, respectively. However, topic 7 was positively correlated with inflammatory indicators in the female ROW group but negatively correlated in the AER male group. This intriguing result suggested that gut microbiota and inflammation might have a sport-specific relationship. We need to investigate further to determine the causes of this sport-specific correlation. Specifically, we observed a strong positive association between topic 7 and inflammatory markers, but *Prevotella* (69.78% of topic 7) was not significantly correlated with the inflammatory markers in ROW female group. These results suggested that the correlation between topic 7 and inflammation may reflect the collective response of the microbes it encompasses, rather than the response of a single microbe. For the AER male group, a negative association was observed between topic 7 and inflammatory markers. Certain *Prevotella* species have been linked to inflammation (24), but not all of them are harmful. For example, *Prevotella copri CB7* has been reported to have both beneficial and detrimental effects depending on the context (25). Our data cannot identify which *Prevotella* species are enriched in the AER male group, and the negative correlation observed may be a unique characteristic of the gut microbiota of athletes that requires further investigation. Overall, gut microbiota can reflect specific inflammatory patterns in different types of athletes, and the association information between the microbial community and inflammatory factors could prove highly significant for monitoring the potential inflammation risk and further achieving personalized exercise modulation. In particular, ROW female athletes should pay more attention to the changes in topic 7 and conduct gut microbial modulation and training monitoring, to avoid the risk of inflammation caused by training as much as possible.

Moreover, by combining athletes and non-athletes cohorts, we found that differences in exercise intensity could lead to specific microbial changes, some of which were associated with inflammation. Broadly speaking, different types and intensities of exercise may have distinct impacts on the gut microbes of general people. In addition to exercising regularly, regulating the types and intensities of exercises is equally important for normal people to gain the benefit of exercise.

Our work has focused on gut microbial community profiling and inflammation pattern mining in multi-sport cohorts. Firstly, the results of this cohort study would lay the foundation for future experiments on the modulation of exercise intensity for inflammation control, or to explore probiotics for athletes to adjust their inflammation levels rather than using antibiotics. Further investigations into mechanisms are warranted to validate the findings of this study. Secondly, conducting multi-omic studies, such as metagenomic and metabolomic analysis may further enhance our understanding of how gut microbial communities adjust inflammation when athletes take high-intensity exercise. Finally, samples from a wider variety of sports in the future will considerably expand our understanding of the athlete microbiota.

### Conclusions

In this study, we collected one of the largest sets of gut microbiota samples from athletes across different sports types and examined their specialized gut microbial profiles. We determined the association between microbes and inflammation in athletes, compared the different strengths of association among different types of sports, and explained their possible roles in regulating inflammation. We found that the differences in microbial abundance in hosts with different sports types may lead to specialized inflammation patterns. Additionally, certain microbial subgroups were also found to be associated with dietary information and anaerobic performance, which were also influenced by the types of sports. Furthermore, changes in the abundance of inflammation-related microbiota in healthy individuals were influenced by different exercise intensities. Finally, we found that sex was a non-negligible factor in both microbial changes and the potential risk of inflammation. Collectively, our work has elucidated the complicated association of physical factors, gut microbiota, and inflammations across different types of sports, which could prove highly significant for monitoring the potential gut inflammation risk and further achieving personalized exercise modulation.

## MATERIALS AND METHODS

### Study design and sample collection

Professional aerobics athletes (AER, *n* = 316, female = 117, male = 199, 18 ± 7 years, 24.80 ± 25.46 kg/m^2^), male wrestling athletes (WRE, *n* = 53, 16 ± 4 years, 22.73 ± 10.60 kg/m^2^), and female rowing athletes (ROW, *n* = 174, 15 ± 3 years, 36.03 ± 19.49 kg/m^2^) were selected for fecal sample collection. According to the grade in athletic competition and information regarding technical level obtained from General Administration of Sport of China, we found that these athletes have participated in at least one competition above the province-level. These athletes differ in terms of training patterns, with aerobics focusing on flexibility, rowing on endurance, and wrestling on both endurance and explosive power. Therefore, we can explore the differences in gut microbiota between athletes under different exercise patterns. Additionally, to further evaluate the effects of exercise with different intensities on the gut microbiota, we constructed a non-athlete cohort. The non-athlete cohort consisted of 58 sports college students (PE, female = 26, male = 32, 21 ± 2 years, 26.19 ± 40.67 kg/m^2^), 63 children (CH, female = 37, male = 26, 9 ± 2 years, 15.80 ± 6.78 kg/m^2^), and 57 sedentary individuals (SD, female = 30, male = 27, 21 ± 2 years). The sports college students come from Wuhan Sports University, and their training intensity and athletic performance are lower than those of professional athletes. The children ranged in age from 7 to 11 years, and they started exercising from early childhood (<6 years). The sedentary individuals are computer-related workers who spend all their working hours sitting (>8 h). All the cohorts had no medical issues or received antibiotic treatment in the past 4 months. Fecal samples were collected and stored in sterilized 50 mL tubes, immediately placed on freezer packs, and stored at −80°C.

Dietary factors, physical characteristics, and sports-related indicators were recorded and examined by the questionnaire and professional measurements for the athlete cohort. These factors were divided into five groups: blood measurements, dietary measurements, anthropometrics (basic metabolism), anthropometrics (body composition), and anaerobic measurements. Blood measurements such as leukocyte, lymphocyte ratio, neutrophil count, hematocrit, and hemoglobin were measured by routine blood tests. Dietary measurements such as the level of smoking, drinking, grain, vegetables, fruit, and soy were recorded by the questionnaires. Anthropometrics referred to the combination of various complex factors such as height, weight, age, basal metabolic capacity, fat free weight, protein capacity, body fat mass, and age obtained by the questionnaire; the height and weight were measured using an electronic height tester; others such as fat free weight, protein capacity, and body fat mass were measured with the professional body composition analyzer (X-SCAN PLUS II, Jawon Medical Co., Ltd, South Korea) (26). We divided these indicators into anthropometrics (basic metabolism) and anthropometrics (body composition), the first of which mainly focused on basal metabolism-related indicators and the latter of which described the composition of the body. Anaerobic measurements were measured using MetaLyzer II (Cortex, Leipzig, Germany) (27) and Technogym multipower system D4773L (Technogym, Italy) (28).

### DNA extraction and 16S rRNA gene sequencing

DNA was extracted from fecal samples using the PowerSoil DNA Isolation Kit (MoBio, USA) according to the manufacturer’s instructions. All extracted DNA was dissolved in Tris-EDTA buffer and stored at −20°C. DNA concentration quantification was performed with a Qubit 2.0 Fluorometer (Invitrogen, Carlsbad, CA, USA), and DNA quality assessment was performed with 0.8% agarose gels. The V3–V4 hypervariable region of the 16S rRNA gene was sequenced for each sample and we used 5–50 ng of DNA as a template for amplifying the V3–V4 amplicon using the forward primer (5ʹ-CCTACGGRRBGCASCAGKVRVGAAT-3ʹ) and reverse primer (5ʹ-GGACTACNVGGGTWTCTAATCC-3ʹ). The sequencing library was constructed using the MetaVxTM Library Preparation kit (Genewiz, Inc., South Plainfield, NJ, USA) via adding indexed adapters to the ends of 16S rDNA amplicons in limited cycle PCR. DNA libraries were verified and quantified by an Agilent 2100 Bioanalyzer (Agilent Technologies, Palo Alto, CA, USA) and Qubit 2.0 (Applied Biosystems, Carlsbad, CA, USA). All sequencing reactions were performed on the Illumina MiSeq (San Diego, CA, USA) platform using a paired-end sequencing strategy.

### 16S rRNA gene sequence data process

The raw multiplexed-paired-end sequences were firstly input to QIIME2 (version 2020.11.0) (29) and were demultiplexed using “qiime cutadapt demux-paired” with “--p-error-rate 0.” The primers of demultiplexed sequences were then trimmed using “qiime cutadapt trim-paired” with “--p-match-adapter-wildcards--p-match-read-wildcards--p-discard-untrimmed.” The trimmed sequences were quality-controlled using “qiime dada2 denoise-paired” with “--p-trunc-len-f 270--p-trunc-len-r 230--p-n-threads 20--p-min-fold-parent-over-abundance 4.” The dada2-produced representative sequences were taxonomically annotated using “qiime feature-classifier classify-sklearn” against the V3–V4 region of the Silva 138 database (30). The taxonomic annotations were integrated into the feature table using “qiime taxa collapse.” For beta diversity analysis, the dada2-produced feature table was rarefied to 8,000 reads per sample using “qiime feature-table rarefy” with “--p-sampling-depth 8,000” based on the curve plateaus of the alpha diversity.

### Statistical analysis

#### Microbial diversity

Statistical analysis was conducted mainly using the R platform (http://www.r-project.org/). Alpha diversity was quantified by the Shannon diversity that was calculated using the function “diversity” of the R package “vegan” (version 2.6-2) (31). Mann-Whitney-Wilcoxon test was used to calculate the statistical significance (*P* values) of the differences in alpha diversity between groups. Beta diversity was quantified by the Bray-Curtis dissimilarity that was calculated using the function “vegdist” of the R package “vegan” (version 2.6-2) (31). Principal coordinates analysis (PCoA) based on Bray-Curtis distances was applied to the samples and visualized by the R package “ggplot2” (version 3.3.6) (32). Mann-Whitney-Wilcoxon test was used to calculate the statistical significance of the sample separation between groups against the PCo1 and PCo2 axes. Permutational analysis of variance (PERMANOVA) on the Bray-Curtis distances with 9,999 permutations was used to test the associations of the phenotype with both microbiome composition and function. PERMANOVA (33) was carried out using the “adonis” function in the R package “vegan” package (version 2.6-2) (31).

#### Prediction model based on gut microbiota to distinguish different athlete types

Random Forest models were generated based on microbial compositions to differentiate three athlete types using the R package “randomForest” (version 4.7-1.1) (34). The data set was randomly divided into the training set (40%) and the testing set (60%). Function “trainControl” in R package “caret” was used to perform 10 repeats of 10-fold cross-validation. Function “train” in R package “caret” was used to fit models over different tuning parameters to determine the “mtry” for Random Forest algorithm. Gini coefficients were used to measure how each variable contributed to the homogeneity of the nodes and leaves in the resulting Random Forest. The receiver operating characteristic (ROC) curve was generated to evaluate the performance of the prediction model.

#### Co-occurrence network analysis

Co-occurrence network analysis was based on the Spearman correlations between genera. The co-occurrence relationship between the two genera was accepted if the Spearman correlation coefficient was greater than 0.55 or less than −0.55 (calculated by R function “cor”) and *P* < 0.05 (calculated by R function "cor.test”). Cytoscape (version 3.9.1) (35) was used to visualize the microbial networks.

#### Latent Dirichlet allocation and phenotype-subgroup associations

Latent Dirichlet allocation (LDA), a Bayesian probabilistic generative model, was used to reveal latent structure present in gut microbial data, as previously described (16). Genera subgroups were identified by LDA and their correlations with phenotype including blood measurements, dietary measurements, anthropometrics (basic metabolism and body composition), and anaerobic measurements were assessed separately using Dirichlet regression models by R package “DirichletReg” (version 0.7.1) (36). To remove the sex effects on gut microbiota or the phenotypic data, we grouped the samples based on sex and the athlete type into four comparison groups including AER males versus WRE males and AER females versus ROW females. The covariates age and body mass index were adjusted in the regression analysis. *P* values for all associations were adjusted using a false discovery rate (FDR), and a significant threshold was FDR < 0.001.

#### Prediction of functional composition and regression analysis

Phylogenetic Investigation of Communities by Reconstruction of Unobserved States (PICRUSt, version 1.0.0-dev) (37) was applied to profile the functional composition of microbial communities based on the high-quality of 16S rRNA gene according to the manual of PICRUSt. The functional trait abundances were determined using the KEGG database (version 66.1, 1 May 2013) (38). Regression analysis of microbiota in topic 7 and inflammatory indicators was performed using R function “cor.test.”

## Data Availability

Sequencing data are available in the Genome Sequence Archive (GSA) section of National Genomics Data Center (project accession number CRA007901).

## SUPPLEMENTAL MATERIAL

Fig. S1 msystems.00259-23-s0001.pdf Comparisons of gut microbiota profiles in multi-sport and sedentary populations.

Fig. S2 msystems.00259-23-s0002.pdf Distribution of topics across the MS cohorts.

Fig. S3 msystems.00259-23-s0003.pdf Identification of gut microbial covariates.

Fig. S4 msystems.00259-23-s0004.pdf Distribution of inflammatory indicators and microbiota related with inflammation.

Fig. S5 msystems.00259-23-s0005.pdf Taxonomical biomarkers in athletes and non-athletes.

Fig. S6 msystems.00259-23-s0006.pdf The distribution of microbiota in topic 7 in athletes and non-athletes.

Table S1 msystems.00259-23-s0007.xlsx Phenotype statistics for athletes.

Table S2 msystems.00259-23-s0008.xlsx Comparisons of taxonomic abundances between different types of sport.

Table S3 msystems.00259-23-s0009.xlsx The each sample's gut microbiota described by a unique composition of 10 topics.

Table S4 msystems.00259-23-s0010.xlsx The probability composition of the genus for each topic.

ASM does not own the copyrights to Supplemental Material that may be linked to, or accessed through, an article. The authors have granted ASM a non-exclusive, world-wide license to publish the Supplemental Material files. Please contact the corresponding author directly for reuse.
