# Supplementary material for: Gut microbiota and inflammation patterns for specialized athletes: a multi-cohort study across different types of sports
Source: mSystems. 2023 Jul 27;8(4):e00259-23. doi: 10.1128/msystems.00259-23 (PMC10470055; doi:10.1128/msystems.00259-23)
Supplement: Fig. S1 — Comparisons of gut microbiota profiles in multi-sport and sedentary populations. [file msystems.00259-23-s0001.pdf]

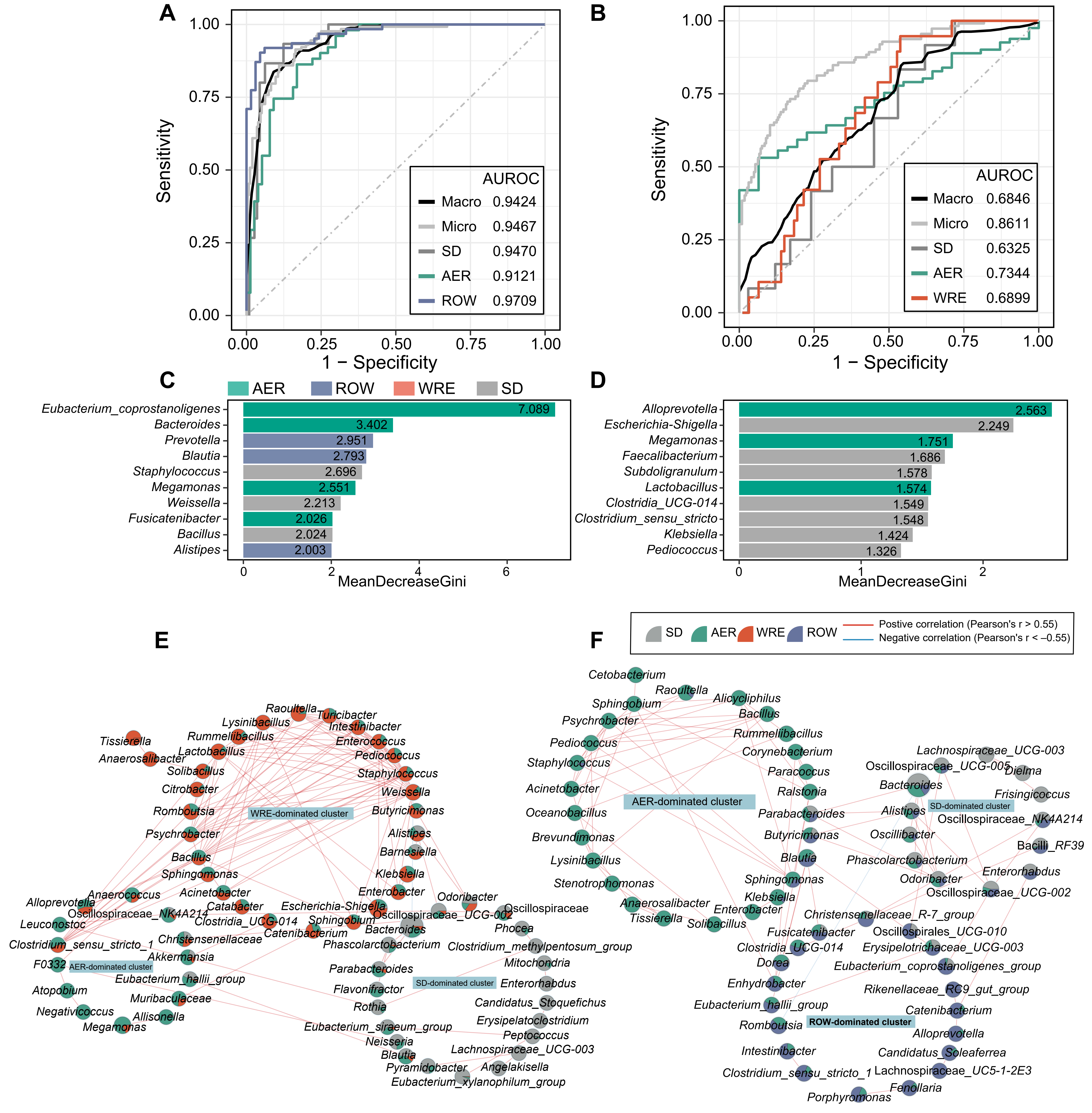

FIG S1. Comparisons of gut microbiota profiles in multi-sport and sedentary populations. (A-B) The ROC curves of the Random Forest classification in types of cohort using microbial genera as features, with AUROC displayed in the female cohort (A) and the male cohort (B). (C-D) The top ten genus contributed to the Random Forest classification in the female cohort (C) and the male cohort (D). (E–F) The networks of genera were constructed in female (E) and male cohorts (F) respectively. Each circle represents a genus with the size representing relative abundance, and the color representing its enrichment in the group. The edges indicate significant Spearman correlations between genus (p<0.05 and absolute value of SCC>0.55). Red edges indicate positive correlations and blue edges indicate negative correlations. SCC, Spearman correlation coefficient.
