# Supplementary material for: Gut microbiota and inflammation patterns for specialized athletes: a multi-cohort study across different types of sports
Source: mSystems. 2023 Jul 27;8(4):e00259-23. doi: 10.1128/msystems.00259-23 (PMC10470055; doi:10.1128/msystems.00259-23)
Supplement: Fig. S2 — Distribution of topics across the MS cohorts. [file msystems.00259-23-s0002.pdf]

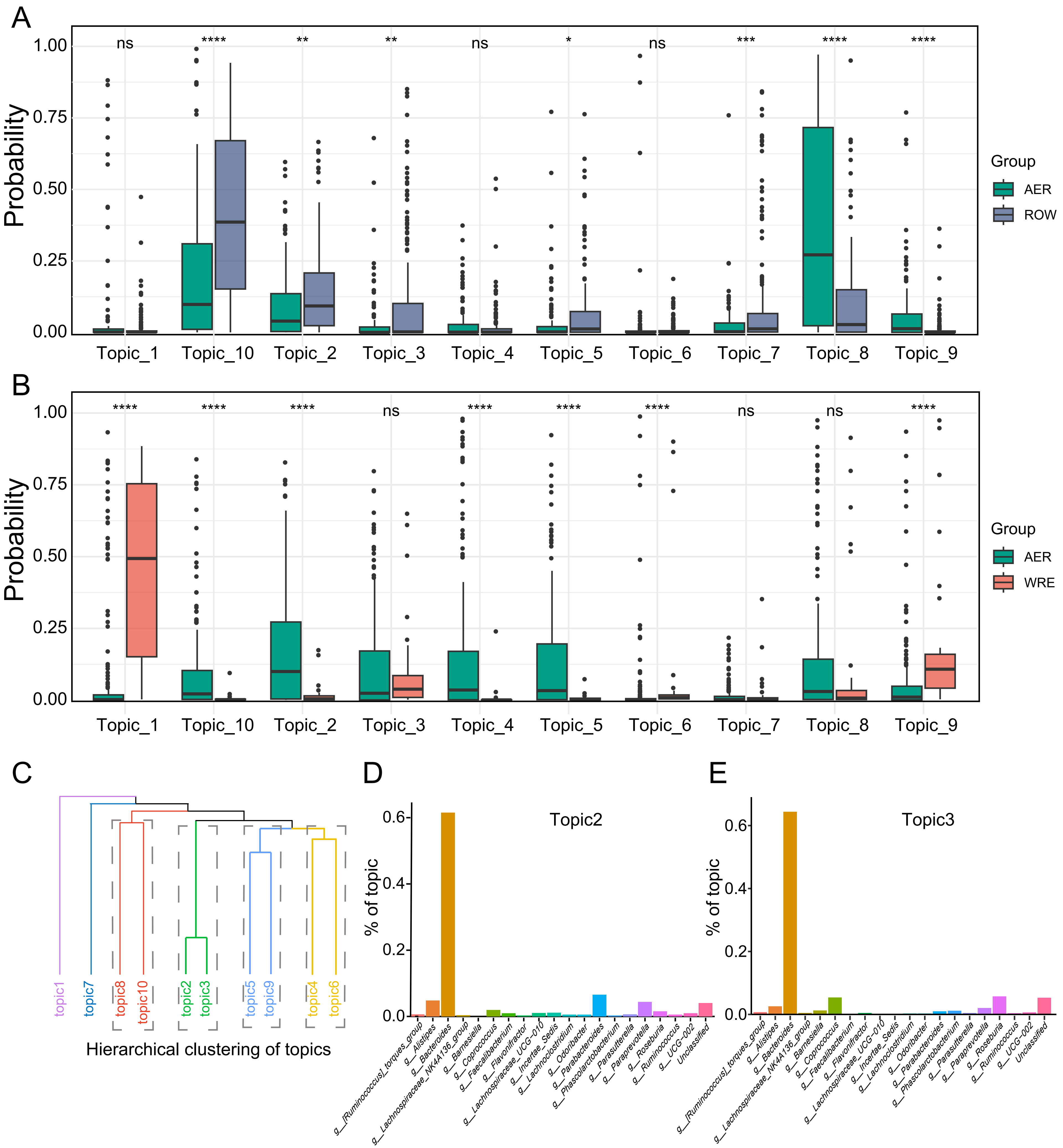

FIG S2. Distribution of topics across the MS cohorts. Each sample's gut microbiota described by a unique composition of 10 topics, topics distribution of samples in MS-female (A) and MS-male cohorts (B) respectively. (C) Hierarchical clustering tree constructed from similarities in the microbiome composition of topics, with each cluster represented by the same color. (D-E) The top 20 genera with the highest microbial possibility in the topic2 (D) and topic3 (E). "*": $P < 0.1$; "**": $P < 0.05$; "***": $P < 0.01$; ns: not significant.
