# Supplementary material for: Gut microbiota and inflammation patterns for specialized athletes: a multi-cohort study across different types of sports
Source: mSystems. 2023 Jul 27;8(4):e00259-23. doi: 10.1128/msystems.00259-23 (PMC10470055; doi:10.1128/msystems.00259-23)
Supplement: Fig. S3 — Identification of gut microbial covariates. [file msystems.00259-23-s0003.pdf]

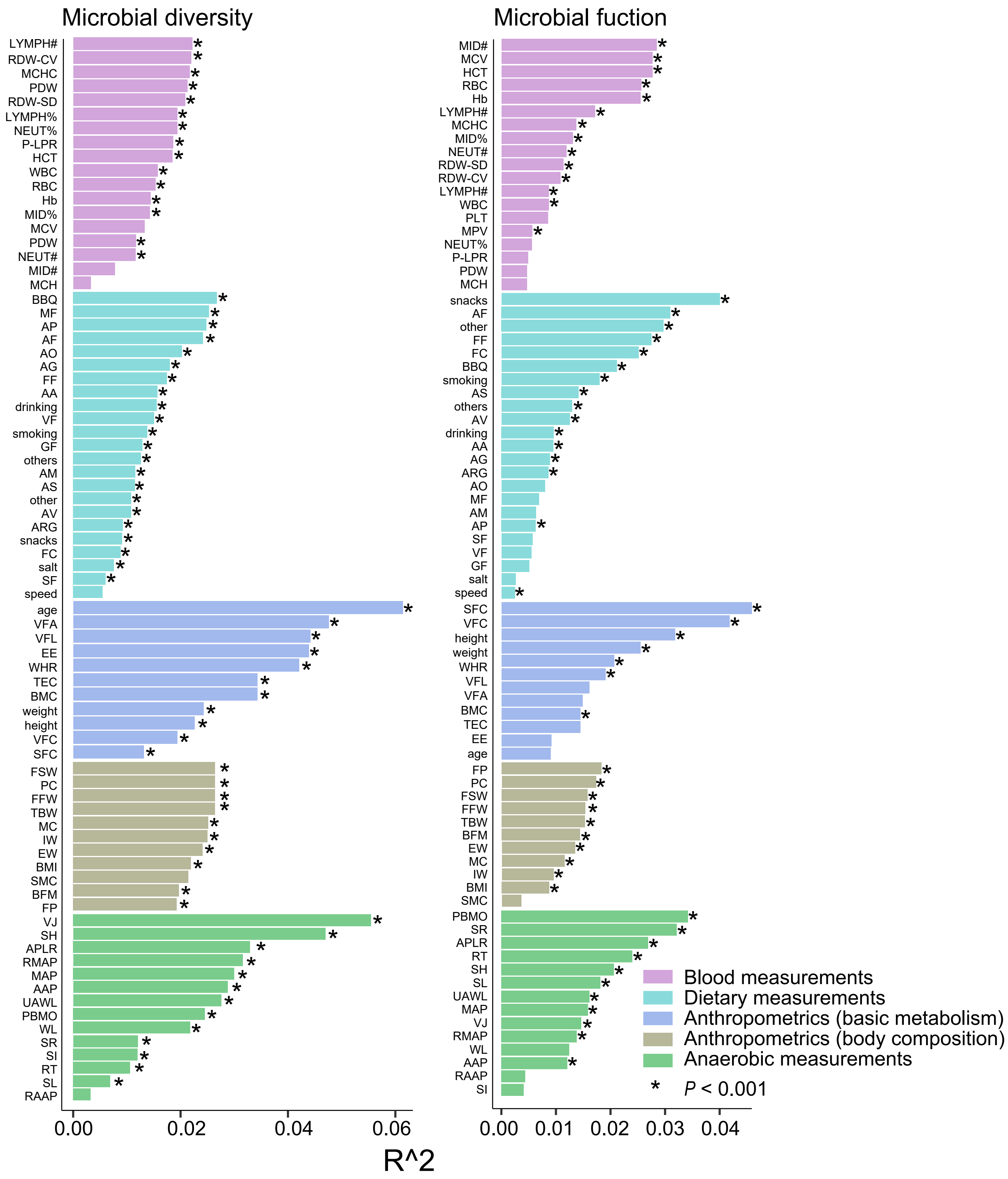

FIG S3. Identification of gut microbial covariates. Bar plot represents explained variance for inter-individual variation in microbial composition and functional composition by permutation analysis of variance (Based on the Bray-Curtis distances), and "*" indicates P < 0.001.
