# Supplementary material for: Gut microbiota and inflammation patterns for specialized athletes: a multi-cohort study across different types of sports
Source: mSystems. 2023 Jul 27;8(4):e00259-23. doi: 10.1128/msystems.00259-23 (PMC10470055; doi:10.1128/msystems.00259-23)
Supplement: Fig. S4 — Distribution of inflammatory indicators and microbiota related with inflammation. [file msystems.00259-23-s0004.pdf]

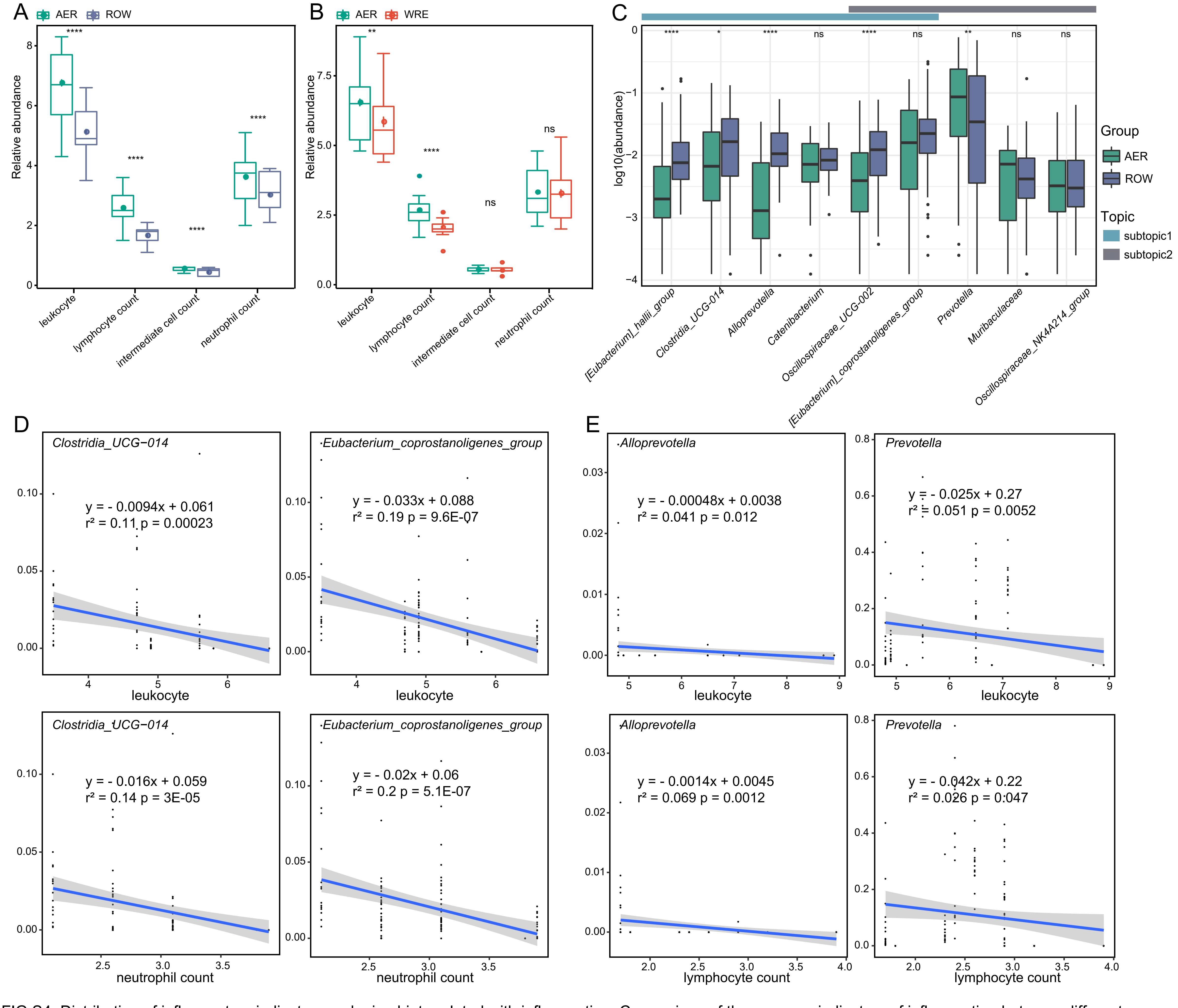

FIG S4. Distribution of inflammatory indicators and microbiota related with inflammation. Comparison of the common indicators of inflammation between different types of sports in the MS-female cohort (A) and MS-male cohort (B). The points indicate the mean relative abundance. (C) The relative abundance of microbiota in topic 7 in male AER and female ROW. Microbiota in topic 7 can be divided into two subtopics according to their enrichment in the correlation network. "*": $P < 0.1$; "**": $P < 0.05$; "***": $P < 0.01$; "****": $P < 0.001$; ns: not significant. (D-E) Regression analysis of microbiota in topic 7 and inflammatory indicators. Microbial and inflammatory indicators showed a significant consistent correlation in the ROW female group (D) and the AER male group (E). No significant associations were not shown in the figure.
