# Supplementary material for: Gut microbiota and inflammation patterns for specialized athletes: a multi-cohort study across different types of sports
Source: mSystems. 2023 Jul 27;8(4):e00259-23. doi: 10.1128/msystems.00259-23 (PMC10470055; doi:10.1128/msystems.00259-23)
Supplement: Fig. S5 — Taxonomical biomarkers in athletes and non-athletes. [file msystems.00259-23-s0005.pdf]

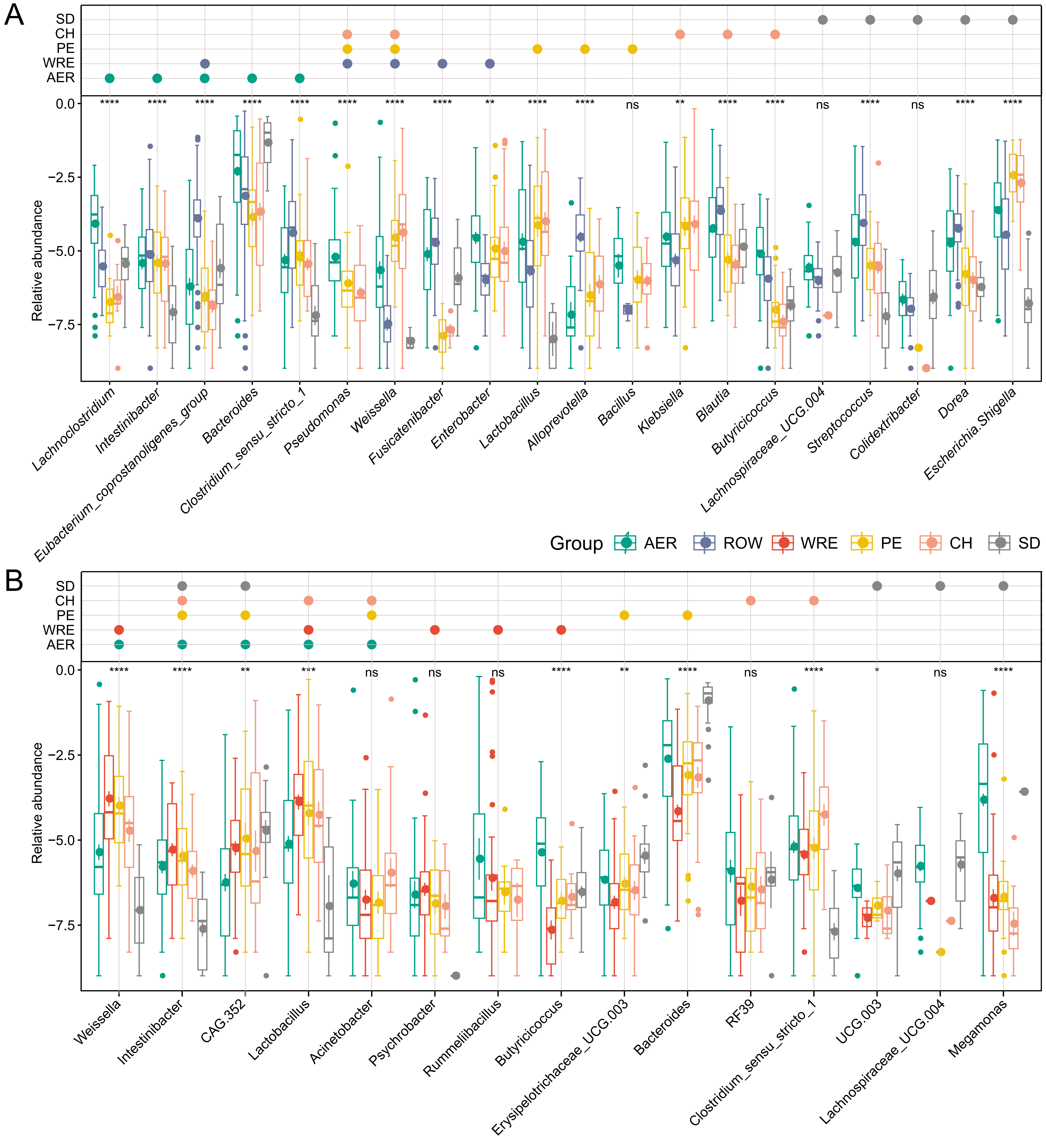

FIG S5. Taxonomical biomarkers in athletes and non-athletes. The top five genera that contributed to the classification accuracy of each group respectively from the random forest model, and the logarithm of relative abundance distribution of these genera in the MS-female cohort (A), as well as in the MS-male cohort (B). The above point represents the bacteria on the x-axis which are the biomarkers of the group, and for each type of cohort, the top five genera contributing to the classification accuracy were selected as biomarkers, i.e. there were 5 points.
