# Supplementary figures and images for: Gut microbiota and inflammation patterns for specialized athletes: a multi-cohort study across different types of sports
Source: mSystems. 2023 Jul 27;8(4):e00259-23. doi: 10.1128/msystems.00259-23 (PMC10470055; doi:10.1128/msystems.00259-23)

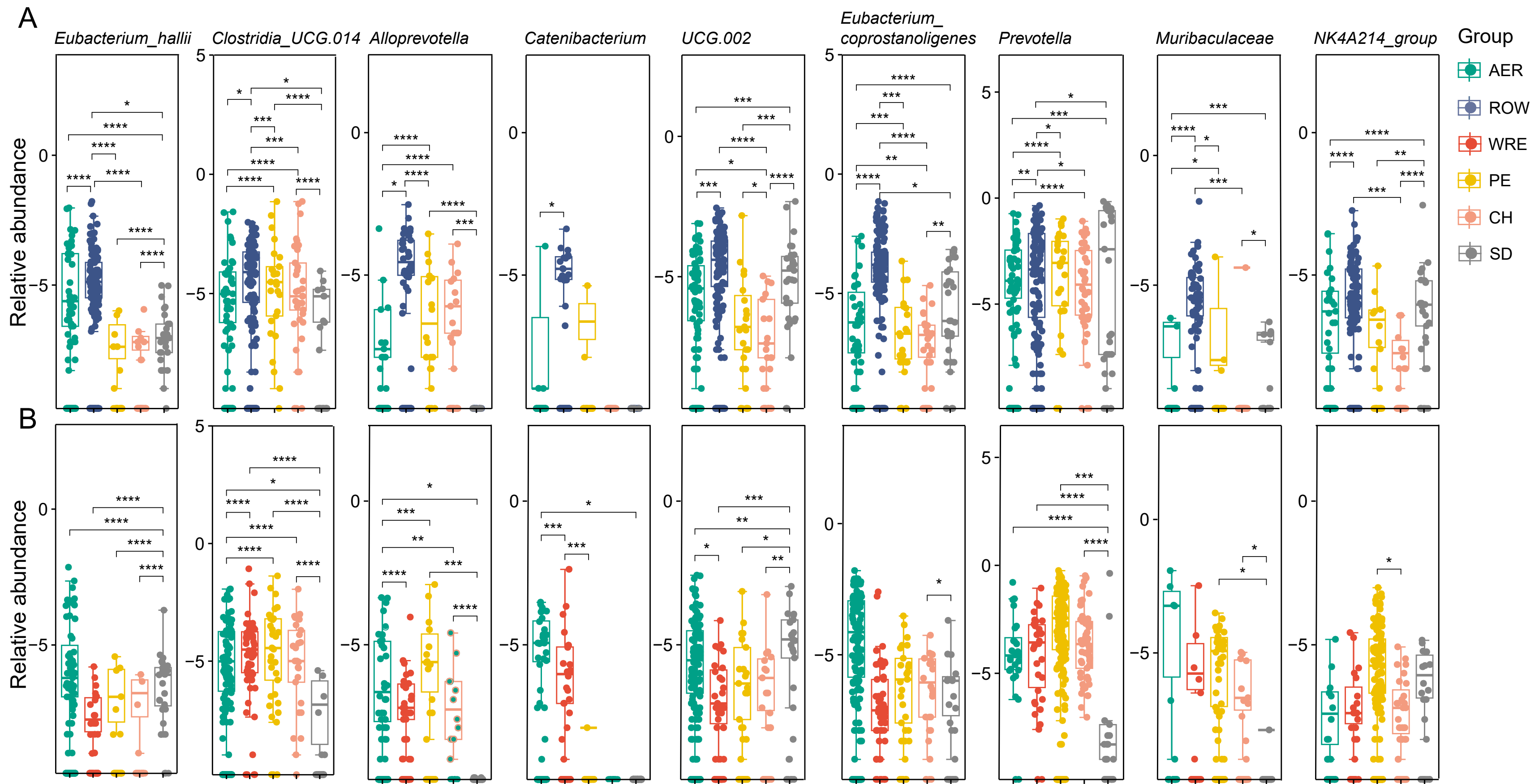

Supplement: Fig. S6 — The distribution of microbiota in topic 7 in athletes and non-athletes. [file msystems.00259-23-s0006.pdf]
